# Public health round-up

**DOI:** 10.2471/BLT.19.010319

**Published:** 2019-03-01

**Affiliations:** https://www.who.int/news-room/detail/28-09-2017-who-statement-on-philip-morris-funded-foundation-for-a-smoke-free-world

**Vaccination against Ebola**

Health workers during an Ebola vaccination campaign in Mangina, North Kivu, Democratic Republic of Congo in 2018.

**Figure:**
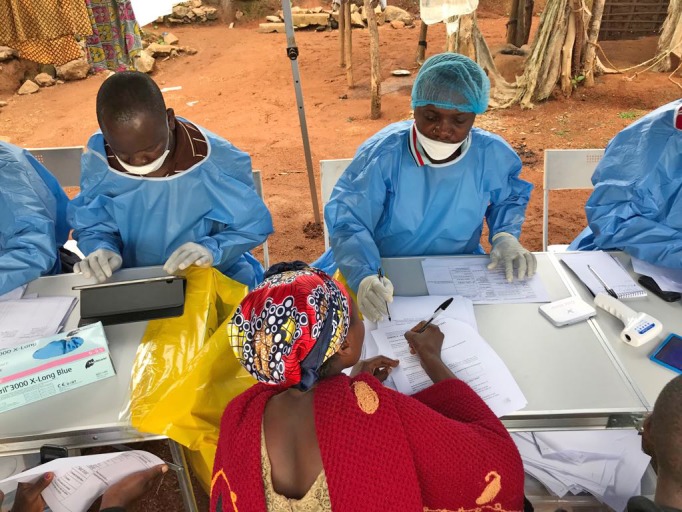
©WHO/Nyka Alexander

## Ebola vaccination in South Sudan

The Ministry of Health of South Sudan has started vaccinating health workers and other front-line responders against Ebola virus disease in high-risk areas bordering the Democratic Republic of the Congo, where an Ebola outbreak began in August 2018.

Vaccination in South Sudan began in Yambio, Gbudue State on 28 January. Health workers in the towns of Tombura, Yei and Nimule as well as the capital city, Juba, were also scheduled to receive the vaccine.

South Sudan received 2160 doses of the Ebola vaccine (rVSV-ZEBOV) from the vaccine developer, Merck. The vaccine offers protection against the Zaire strain of the virus, the one affecting the Democratic Republic of the Congo at present.

The South Sudan Ministry of Health is being supported by the World Health Organization (WHO), Gavi, the Vaccine Alliance, United Nations Children’s Fund, the US Centers for Disease Control and Prevention and other partners.

In the Democratic Republic of the Congo, as of 12 February, 819 cases (758 confirmed and 61 probable) of Ebola virus infection had been reported, including 516 deaths. Katwa and Butembo health zones remained the epicentres of the outbreak, with smaller clusters continuing to occur across a geographically dispersed area. Neighbouring countries have not yet reported any cases, but preparedness measures to fight the spread of the disease are of vital importance.

https://afro.who.int/news/south-sudan-vaccinates-health-workers-against-ebola

## WHO rejects collaboration with Philip Morris-funded foundation

WHO Director-General Tedros Adhanom Ghebreyesus rejected a collaboration proposal from the Foundation for a Smoke-Free World, funded by Philip-Morris International.

The foundation proposed that WHO “review and consider how best to work with the foundation to facilitate a rapid reduction in the use of lethal cigarettes” in an open letter to the WHO Executive Board which met between 24 January and 1 February.

Speaking to the Executive Board, the Director-General stated that WHO would not partner with the foundation and recommended that Member States and and their counterparts also refuse to do so.

“WHO will NOT partner with Philip Morris-funded foundation for a Smoke-Free World nor any other group funded by the tobacco industry. Governments and the public health community should follow this lead” was the message tweeted by @WHO, shortly after his statement.

The statement repeats the position articulated by WHO in 2017, when the foundation was created.

The foundation claims to provide grants for “medical, agricultural, and scientific research to end smoking and its health effects and to address the impact of reduced worldwide demand for tobacco.” Philip Morris is introducing a new portfolio of products, including heated tobacco products, into many markets. Research on such products is funded by the foundation.

The WHO Framework Convention on Tobacco Control’s (WHO FCTC) Secretariat has been similarly forthright in its rejection of the foundation, stating that it is a clear attempt to breach the WHO FCTC by interfering in public policy “aimed at damaging the treaty’s implementation, particularly through the foundation’s contentious research programmes.”

## Lassa fever in West Africa

Five countries – Benin, Guinea, Liberia, Nigeria and Togo – are reporting outbreaks of Lassa fever. The largest of these outbreaks was affecting 19 states in Nigeria as of 3 February.

The Nigeria Centre for Disease Control declared an outbreak of Lassa fever on 22 January 2019, and as of 3 February, had reported 275 confirmed positive cases in 19 states, including 57 deaths.

This represents slightly over a third of the total cases for last year, when Nigeria experienced its worst outbreak of Lassa fever to date. A total of 12 cases had been confirmed as of 3 February in Benin, Guinea, Liberia and Togo, including two deaths, with more suspected cases being investigated.

“We are concerned by the high number of cases so early in the Lassa fever season, which is expected to last another four more months,” said Dr Ibrahima Socé Fall, Regional Emergencies Director at WHO Regional Office for Africa.

Lassa fever outbreak response is focused on early detection and confirmation of suspected cases, providing optimal supportive care and ensuring infection prevention and control measures in designated health-care facilities in the affected states.

WHO is supporting the outbreak response coordination, enhanced surveillance, epidemiological analysis and risk communication in all five countries. The organization is also mobilizing experts to support case management and infection prevention and control.

Lassa fever is an acute viral haemorrhagic illness that occurs predominantly in West Africa, after human exposure to the urine or faeces of infected rats. More than 80% of Lassa fever cases are caused by rodent-to-human transmission of the virus. Prevention relies on promoting good “community hygiene” to discourage rodents from entering homes.

https://afro.who.int/news/who-supports-five-countries-fight-lassa-fever-outbreaks

## Funding for neglected disease R&D rises

Global funding for basic research and product development for neglected diseases reached US$ 3.5 billion in 2017 according to the latest *G-FINDER survey, *which has been tracking**global investment in innovative research and development for neglected diseases since 2007.

It was the highest level recorded by the survey apart from the US$ 3.7 billion reported in 2015, which was boosted by an investment surge in research and development for viral haemorrhagic fevers.

Investment in 2017 grew by US$ 232 million compared to 2016. This is the largest increase in both relative and absolute terms since 2008.

Investment growth was primarily driven by the governments of Germany, India, the United Kingdom of Great Britain and Northern Ireland, and by the European Commission.

Investment continues to be heavily skewed towards HIV, tuberculosis and malaria which collectively received 70% of global neglected disease research and development funding (US$ 2.5 billion) in 2017. Several high burden diseases are still neglected; for example, funding for dengue decreased by 28%, dropping to just US$ 32 million.

Despite increased overall funding, no national government met the recommendation of the *WHO Global Strategy and Plan of Action on Public Health, Innovation and Intellectual Property* that Member States dedicate at least 0.01% of their gross domestic product to research into the health needs of people in low- and middle-income countries.

https://www.policycuresresearch.org/g-finder-2018/

## New audio device standard aims to prevent hearing loss

WHO and the International Telecommunication Union (ITU) issued a new international standard for the manufacture and use of personal audio devices, including smartphones and audio players, to make them safer for listening.

Just under 50% of people aged 12-35 years – or 1.1 billion young people – are at risk of hearing loss due to prolonged and excessive exposure to loud sounds.

Launched 12 February, the standard was developed under WHO’s “Make Listening Safe” initiative which seeks to improve listening practices especially among young people, both when they are exposed to music and other sounds at noisy entertainment venues, and as they listen to music through their personal audio devices.

The standard was developed by experts from WHO and ITU over a two-year process drawing on the latest evidence and consultations with a range of stakeholders, including experts from government and industry, consumers and civil society.

https://www.who.int/news-room/detail/12-02-2019-new-who-itu-standard-aims-to-prevent-hearing-loss-among-1.1-billion-young-people

## Libya health system appeal

WHO and health partners are appealing for US$ 43.5 million to provide life-saving interventions for 388 000 people inside Libya affected by ongoing conflict there.

Launched 11 February, the appeal is an attempt to support a health system that has been undermined by years of conflict.

“Many health facilities are fully or partially closed, limiting access to health-care services to a population suffering from challenges from the 8-year conflict,” said Dr Syed Jaffar Hussain, WHO Representative in Libya.

An assessment of health service availability and readiness by WHO and the Ministry of Health in 2017 showed that 17.5% of hospitals, 20% of primary health care facilities and 18 specialized hospitals are damaged or destroyed.

Health facilities that remain functional are at continued risk of attack, with more than 41 attacks targeting health workers and facilities across the country reported by WHO in 2018–2019. http://www.emro.who.int/lby/libya-news/us-435-million-needed-to-provide-life-saving-health-aid-in-libya-in-2019.html

**Cover photo**

Five year-old child standing in front of the family ger (traditional circular dwelling) in Khuvsgul province, northern Mongolia, February, 2017.

**Figure:**
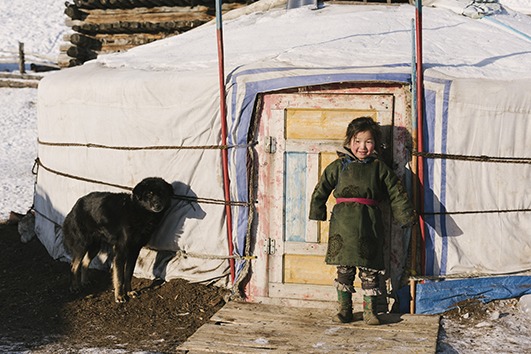
IFRC/ Benjamin Suomela

**Looking ahead**

11 – 22 March - UN Commission on the Status of Women, Geneva

24 March - World TB Day

11 – 13 April - Fair Pricing Forum for Medicines, Geneva

23 – 24 April - The Food and Agriculture Organization, World Health Organization, and World Trade Organization International Forum on Food Safety and Trade, Geneva

